# 

**Published:** 1892-07

**Authors:** 


					﻿VOL. XII.
JULY, 1892.
No. 7.
THE
HOMEOPATHIC PHYSICIAN,
A Monthly Journal of Homoeopathic Materia Medica
and Clinical Medicine.
“ He is a freeman whom truth makes free, and all are slaves beside."
“Seek the Truth: come whence it may, cost what it will."
EDITED AND PVBI.ISIIED BY
WALTER XI. JAMES, XI. D.
PHILADELPHIA :
No. 1125 SPRL’CK 8TREET.
I/O 2ST D O IT :
ALFRED HEATH A CO., Sole Agents for Great Britain,
114 Ebury Street, Eaton Square.
O“Tear/p Subscription, $2.SO. invariably in advance. Single numbers, 2S els.
Entered at the Philadelphia Peet-OIBce aa Seoood Claai Mall Mauer.
				

